# Prognostic value of the neutrophil-to-lymphocyte ratio in acute organophosphorus pesticide poisoning

**DOI:** 10.1515/biol-2021-0069

**Published:** 2021-07-15

**Authors:** Yuhang Mu, Boqi Hu, Nan Gao, Li Pang

**Affiliations:** Department of Emergency, First Hospital of Jilin University, No. 1 Xinmin Road, Changchun, 130021, China; Department of Radiology, China-Japan Union Hospital of Jilin University, Changchun, 130033, China; Department of Emergency, Third Clinical Hospital of Changchun Traditional Chinese Medicine University, Changchun, 130117, China

**Keywords:** AOPP, NLR, public health, risk factor, prognosis

## Abstract

This study investigates the ability of blood neutrophil-to-lymphocyte ratio (NLR) to predict acute organophosphorus pesticide poisoning (AOPP). Clinical data of 385 patients with AOPP were obtained within 24 h of admission, and NLR values were calculated based on neutrophil and lymphocyte counts. The patients were divided into two groups – good and poor – based on prognosis. Poor prognosis included in-hospital death and severe poisoning. The factors affecting prognosis were analyzed by logistic regression analysis, and the prognostic value of NLR was evaluated using the area under the receiver operating characteristic curve (AUC). Univariate logistic regression analysis showed that NLR levels, serum cholinesterase, and creatinine levels were good predictors of AOPP. Multivariate logistic regression analysis showed that high NLR was an independent risk factor for severe poisoning (adjusted odds ratio [AOR], 1.13; 95% CI, 1.10–1.17; *p* < 0.05) and in-hospital mortality (AOR, 1.07; 95% CI, 1.03–1.11; *p* < 0.05). NLR values >13 and >17 had a moderate ability to predict severe poisoning and in-hospital mortality, respectively (AUC of 0.782 [95% CI, 0.74–0.824] and 0.714 [95% CI, 0.626–0.803], respectively). Our results show that high NLR at admission is an independent indicator of poor prognosis in AOPP and can be used to optimize treatment and manage patients.

## 1 Introduction

Organophosphate pesticides (OPs) have been widely used for controlling agricultural and forestry pests worldwide since the 1930s because of their strong insecticidal effect and low cost. However, acute OP poisoning (AOPP) is a significant problem and a potential cause of mortality in the developing world, especially in Asian countries, because of the lack of regulation and easy availability of these products [1].

AOPP progresses rapidly. Disease severity is evaluated based on symptoms and routine laboratory tests. Typical symptoms include salivation, sweating, pupil constriction, and muscle fibrillation. Critically ill patients present respiratory failure, disturbance of consciousness, shock, and death. However, initial symptoms are not detected in the emergency room in many patients because of prompt first aid.

Some indicators of the severity of AOPP have been identified. For instance, the determination of cholinesterase (ChE) levels in the plasma of AOPP patients is widely used in clinical diagnosis, treatment, and prognosis. In addition, Acute Physiology and Chronic Health Evaluation II (APACHE II) scores are positively correlated with disease severity and serve as a prognostic marker in adult AOPP patients admitted to the ICU [2]. Blood lactate level is a good indicator of perfusion status, and hyperlactatemia is associated with increased case fatality rates [3]. Furthermore, caspase [4], serum amylase [5], and other markers [6,7,8] have been used to predict the prognosis of patients with OP poisoning. However, these scoring systems are complex and not widely applied in clinical practice because of hospital limitations. Therefore, it is crucial to identify simple, reliable, and easily obtainable clinical markers to assess the severity and prognosis of patients with AOPP.

The neutrophil-to-lymphocyte ratio (NLR) is an independent predictor of short- and long-term mortality in critically ill patients, and measurement is simple, cheap, and fast [9]. Furthermore, the NLR is a powerful prognostic predictor of cardiovascular disease, cancer, acute ischemic stroke, and other diseases [10,11,12,13,14]. This study evaluates the prognostic value of NLR in AOPP.

## 2 Materials and methods

### 2.1 Study design and data collection

This retrospective cohort study was conducted at First Hospital of Jilin University from January 1, 2013, to December 31, 2018. A questionnaire containing the following variables was used: age, gender, medical history (hypertension, diabetes, heart failure, chronic obstructive pulmonary disease [COPD], chronic kidney disease [CKD], and liver cirrhosis), blood tests (white blood cell count, hematocrit, platelet count, plasma cholinesterase [pChE], urea nitrogen, serum creatinine, serum amylase, and albumin), and blood gas analysis (pH, partial pressure of oxygen and carbon dioxide, base excess, and lactate) within 24 h after hospital admission. These data were collected by two experienced physicians and transferred to Excel spreadsheets. The data were anonymized.


**Informed consent:** Informed consent has been obtained from all individuals included in this study.
**Ethical approval:** The research related to human use has been complied with all the relevant national regulations, institutional policies, and in accordance with the tenets of the Helsinki Declaration and has been approved by the Research Ethics Committee of the First Hospital of Jilin University.

### 2.2 Diagnosis of AOPP

AOPP was defined as oral exposure to OPs and pChE values lower than 4,300 U/L at admission.

### 2.3 Inclusion and exclusion criteria and outcomes

The inclusion criteria were as follows: age >18 years, oral exposure to OPs, and hospital admission within 24 h after exposure. The exclusion criteria were as follows: exposure to medications or toxic chemicals other than OPs, patients with normal pChE levels, and patients with severe chronic comorbidities, including liver cirrhosis, symptomatic heart failure (New York Heart Association class III or IV), end-stage CKD (on regular hemodialysis), and COPD.

Outcomes were in-hospital mortality and severe AOPP, and the latter was characterized by ventilatory support; hypotension or shock and the need for dopamine or norepinephrine to maintain hemodynamic stability; and cardiac arrest or death.

### 2.4 Statistical analysis

Continuous variables were expressed as medians (IQR) and were compared using the Kruskal–Wallis test. Categorical variables were expressed as numbers (proportions) and were compared using the chi-square test or Fisher’s exact test. The confounding effects of demographic characteristics, medical history, and laboratory test results on severe AOPP and in-hospital mortality were evaluated using univariate logistic regression and backward stepwise multivariate logistic regression. Multicollinearity was checked using variance inflation factors. The prognostic value of NLR in AOPP was analyzed by the area under the receiver operating characteristic curve (AUC). The optimal cutoff for NLR was estimated using the Youden index. A two-tailed *p*-value of less than 0.05 was considered to be statistically significant. All analyses were performed using R software version 3.6.0 (R Foundation for Statistical Computing, Vienna, Austria).

## 3 Results

### 3.1 Baseline characteristics

**Figure 1:**
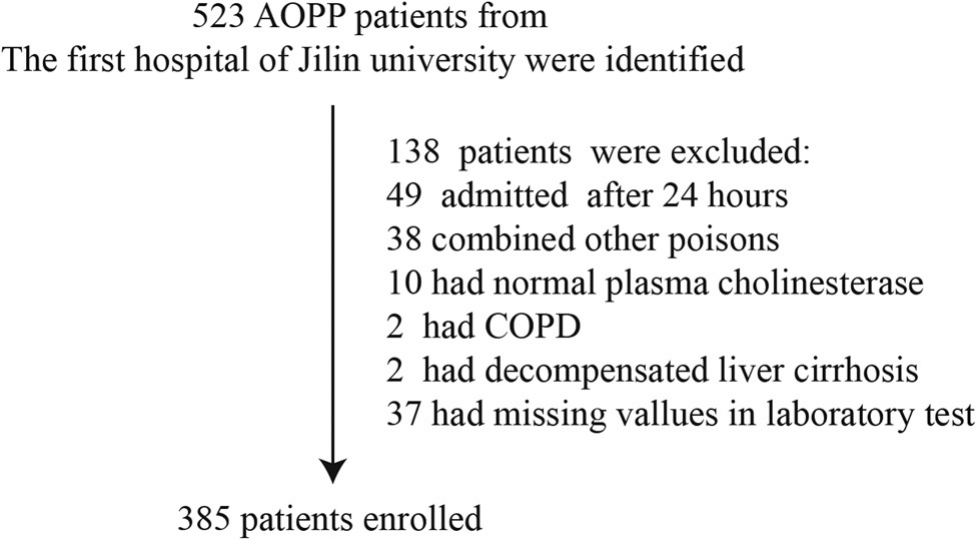
Patient selection.

A total of 523 patients were identified retrospectively from a hospital database. Of whom, 138 (26.4%) were excluded because of exposure to toxic chemicals other than OPs (38, 7.3%), admission after 24 h of exposure (49, 9.4%), normal pChE on admission (10, 1.9%), COPD (2, 0.4%), decompensated liver cirrhosis (2, 0.4%), or missing laboratory data (37, 7.1%). Data from 385 AOPP patients were included in the analysis. The flowchart of patient selection is shown in Figure 1. The percentage of severe cases and overall mortality was 23.6 and 7.3%, respectively. The median NLR was 11.8 (7.35–19.49). Patients were further divided into three groups according to NLR values: <12.0, 12.0–23.9, and ≥24.0. Baseline characteristics according to NLR levels are shown in Table 1. The rate of diabetes, number of neutrophils and lymphocytes, pChE, amylase, pH, lactate, number of patients with AOPP, and in-hospital mortality were significantly different across these three groups (Table 1).

**Table 1:** Baseline characteristics according to the neutrophil-to-lymphocyte ratio

	L-NLR	M-NLR	H-NLR	*p*-value
	(NLR ≤ 12)	(12 < NLR ≤ 24)	(NLR > 24)	
Patients	196	128	61	
Age (years)	42 (33–51)	43 (33–58)	13 (30–60)	0.363
Gender, M/F	100/96	65/63	29/32	0.888
Hypertension, *n* (%)	14 (7.1)	9 (7.0)	6 (9.8)	0.759
Diabetes, *n* (%)	4 (2.0)	8 (6.3)	7 (11.5)	0.009
**Clinical data**				
Neutrophil	8.7 (5.8–12.8)	15.3 (12.3–19.6)	16.9 (14.2–21.7)	<0.001
Lymphocyte	1.5 (1.1–1.8)	0.9 (0.7–2.5)	0.5 (0.4–0.7)	<0.001
pChE	565 (282–1596)	378 (255–1256)	321 (217–776)	0.018
Amylase	91.5 (57.8–176.0)	150 (79.0–317.0)	150 (88.0–330.0)	<0.001
sCr	0.65 (0.55–0.81)	0.68 (0.58–0.85)	0.69 (0.59–0.88)	0.32
pH	7.4 (7.34–3.45)	7.37 (7.29–7.42)	7.35 (7.27–7.41)	<0.001
Lactate	1.3 (0.8–2.4)	1.6 (1.0–3.5)	1.6 (1.0–3.0)	0.03
**Complications**				
Ventilatory support	7 (5.4)	39 (30.5)	32 (52.5)	<0.001
Shock	6 (4.7)	43 (33.6)	31 (50.8)	<0.001
Cardiac arrest	0	1 (0.8)	3 (4.9)	0.006
**Outcomes**				
Severe poisoning	7 (3.6)	50 (39.1)	34 (55.7)	<0.001
Death	4 (2.0)	13 (10.2)	11 (18.0)	<0.001

NLR, neutrophil-to-lymphocyte ratio; pChE, plasma cholinesterase; sCr, serum creatinine.

### 3.2 NLR as indicators of severe poisoning and in-hospital mortality in AOPP patients

**Figure 2:**
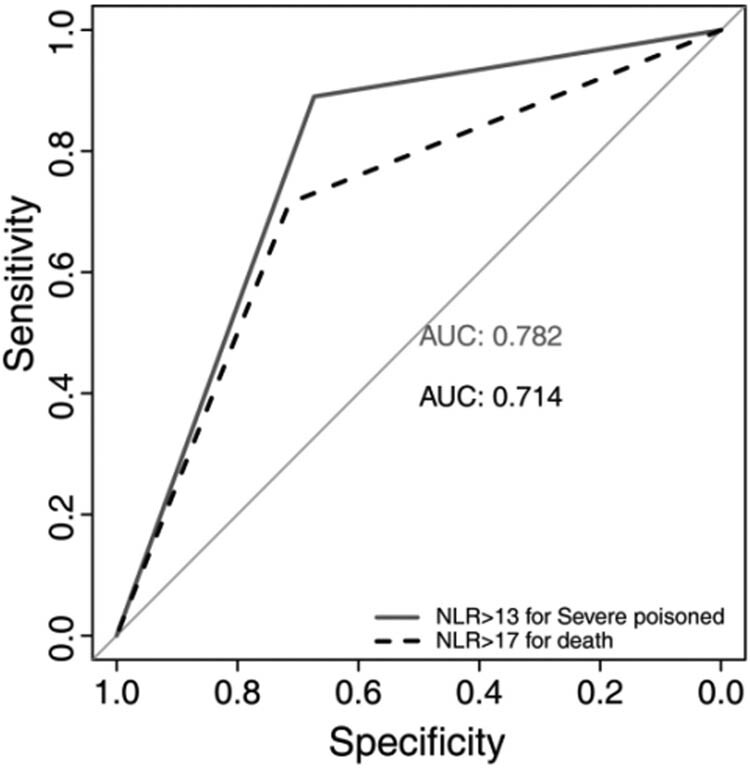
Area under the receiver operating characteristic curve of the neutrophil-to-lymphocyte ratio (NLR) for predicting severe poisoning and in-hospital mortality. NLR with cutoff values.

**Figure 3:**
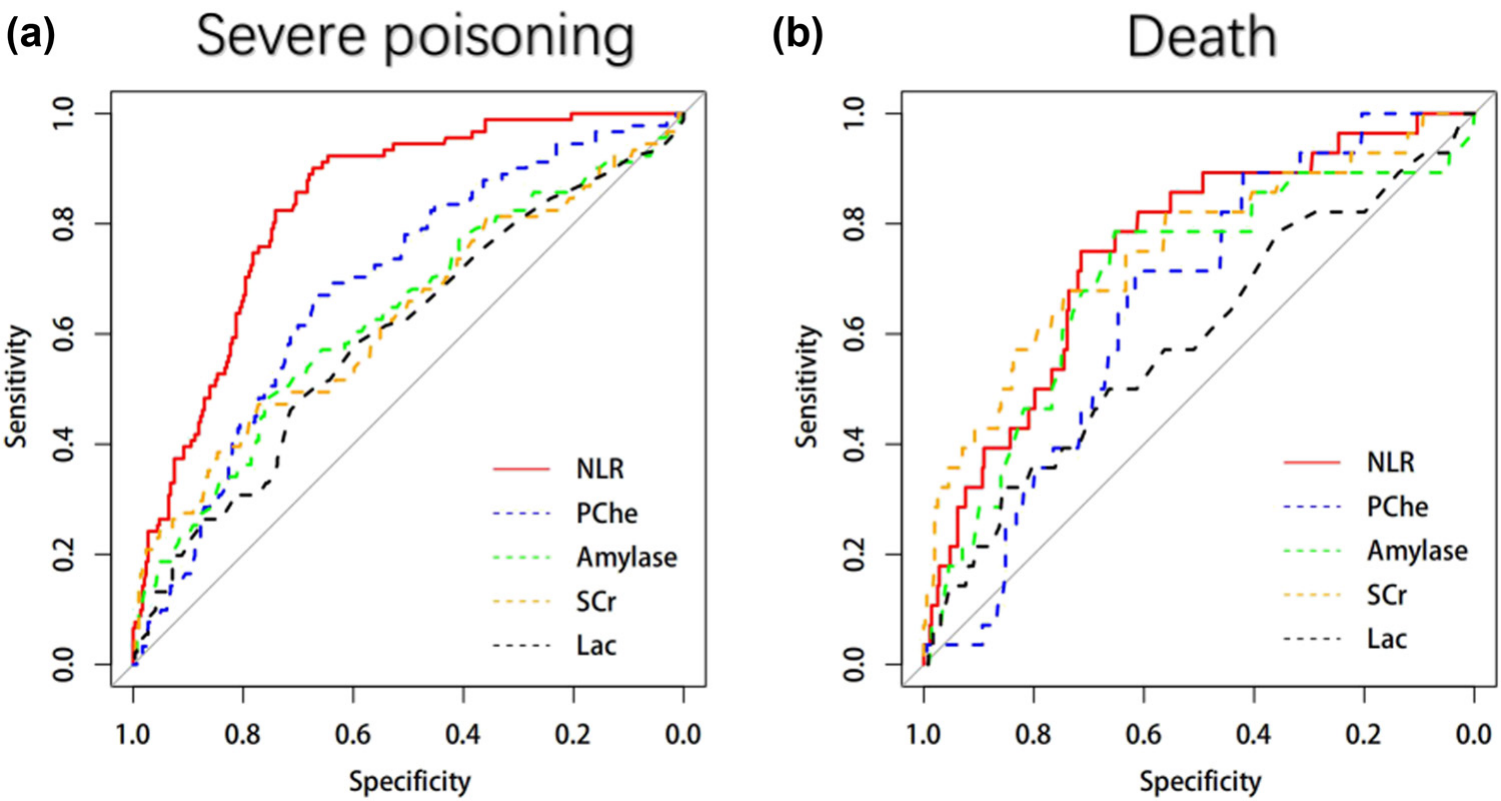
Area under the receiver operating characteristic curve for severe poisoning (a) and death (b).

The results of the univariate logistic regression analysis are shown in Table 2. Variables with *p*-values of less than 0.2 were included in multivariate logistic regression models. After backward stepwise logistic regression, the adjusted odds ratio (AOR) of NLR was 1.13 (1.10–1.17, *p* < 0.001) and 1.07 (1.03–1.11, *p* < 0.001) for severe AOPP and in-hospital mortality, respectively (Table 3). No multicollinearity was detected in the multivariate models (Appendix Table A1). The AUC of NLR for discriminating severe poisoning and in-hospital mortality was 0.83 (95% CI, 0.79–0.89) and 0.75 (95% CI, 0.66–0.84), respectively, and the optimal cutoff for predicting these events was 13 and 17, respectively. The AUC of NLR >13 for discriminating severe poisoning was 0.78 [95% CI, 0.74–0.82], with a sensitivity of 0.89 and specificity of 0.67. The AUC of NLR >17 for discriminating death was 0.71 [95% CI, 0.63–0.80], with a sensitivity of 0.71 and specificity of 0.71 (Figure 2 and Table 4). The AUCs of pChE, amylase, serum creatine, and lactate were also calculated (Figure 3 and Table 4).

**Table 2:** Univariate logistic regression analysis of severe poisoning and in-hospital mortality

Variables	Severe poisoning			Death		
	OR	95% CI	*p*-value	OR	95% CI	*p*-value
Age (years)	1.33	1.14–1.56	<0.001	1.18	0.92–1.51	0.182
Male	1.20	0.75–1.93	0.451	2.2	0.99–5.22	0.06
Hypertension, *n* (%)	2.48	1.11–5.37	0.023	0.94	0.15–3.39	0.935
Diabetes, *n* (%)	3.91	1.53–10.16	0.004	7.22	2.35–20.23	<0.001
Neutrophil	1.13	1.09–1.18	<0.001	1.09	1.03–1.15	0.002
Lymphocyte	0.22	0.12–0.38	<0.001	0.4	0.17–0.84	0.027
NLR	1.13	1.10–1.17	<0.001	1.07	1.01–1.11	<0.001
pChE	0.93	0.90–0.96	<0.001	0.91	0.82–0.97	0.014
Amylase	1.02	1.01–1.03	<0.001	1.02	1.01–1.03	0.002
sCr	1.18	1.09–1.29	<0.001	1.22	1.11–1.36	<0.001
Lactate	1.18	1.06–1.32	0.003	1.14	0.97–1.32	0.082

OR, odds ratio; CI, confidence interval; NLR, neutrophil-to-lymphocyte ratio; pChE, plasma cholinesterase; sCr, serum creatinine.

**Table 3:** Multivariate logistic regression analysis of severe poisoning and in-hospital mortality

Variables	Severe poisoning			Death		
	AOR	95% CI	*p*-value	AOR	95% CI	*p*-value
Age (years)	1.18	0.96–1.45	0.112	NI		
Diabetes, *n* (%)	NI			15.88	3.12–86.46	<0.001
Hypertension, *n* (%)	2.28	0.77–6.58	0.129	0.15	0.01–0.99	0.076
NLR	1.13	1.10–1.17	<0.001	1.07	1.03–1.11	<0.001
pChE	0.94	0.90–0.98	0.002	0.93	0.84–0.99	0.078
Amylase	1.01	1.00–1.02	0.085	NI		
sCr	1.13	1.05–1.25	0.006	1.22	1.10–1.37	<0.001
Lactate	1.17	1.00–1.36	0.042	NI		

AOR, adjusted odds ratio; CI, confidence interval; NI, not included in the model; NLR, neutrophil-to-lymphocyte ratio; pChE, plasma cholinesterase; sCr, serum creatinine.

**Table 4:** Area under the receiver operating characteristic curve for severe poisoning and in-hospital mortality

Variables	Severe poisoning		Death	
	AUC	95% CI	AUC	95% CI
NLR	0.83	0.79–0.89	0.75	0.66–0.84
pChE	0.69	0.62–0.75	0.65	0.57–0.74
Amylase	0.63	0.56–0.70	0.70	0.59–0.81
sCr	0.63	0.56–0.63	0.75	0.64–0.86
Lactate	0.60	0.53–0.67	0.59	0.47–0.71

CI, confidence interval; NLR, neutrophil-to-lymphocyte ratio; pChE, plasma cholinesterase; sCr, serum creatinine.

## 4 Discussion

Pesticide exposure remains a global public health problem [15]. OPs are responsible for the majority of deaths in self-poisoning cases, particularly in rural areas [16]. The estimated annual number of deaths from AOPP worldwide is 100,000 [17]. Despite the availability of specific treatments, mortality can reach more than 20% [18]. Therefore, evaluating the severity of AOPP is essential, and timely and accurate assessments by emergency physicians can help improve treatment efficacy.

This study investigated demographic and clinical parameters associated with the prognosis of AOPP. The rate of severe poisoning and in-hospital mortality was significantly higher in the high NLR group. Multivariate logistic regression analysis showed that NLR on admission was an independent predictor of poor prognosis in patients with AOPP after adjusting for covariates.

Severe AOPP causes failure of multiple organs, and clinical manifestations include respiratory failure, acute myocardial injury, cognitive impairment caused by nervous system injury, and decline in spatial learning ability [19,20,21]. Nonetheless, the molecular mechanisms underlying AOPP are not fully understood and may include the inhibition of cholinesterase activity in the nervous system, resulting in the accumulation of acetylcholine and the dysfunction of the cholinergic system [17] and inhibition of mitochondrial oxidative phosphorylation and mitochondrial membrane disruption [22]. The leakage of electrons in the mitochondrial respiratory chain produces reactive oxygen species (ROS), resulting in oxidative damage, which impairs mitochondrial function, cellular respiration, and energy production, ultimately leading to cell apoptosis and necrosis [23,24].

The clinical variables currently used to predict the severity of AOPP include APACHE II scores [2], plasma cholinesterase activity, plasma amylase [5,25], and blood lactate [3]. However, the clinical usefulness of these variables is limited. APACHE II scores evaluate disease severity based on patient age, medical history, and 12 physiological parameters measured at admission. This scoring system is complex and depends on a subjective assessment of clinical data; thus, it is difficult to apply in emergency settings because of its high complexity. Blood lactate is a marker of AOPP severity and prognosis [7]. In our cohort, blood lactate level was significantly higher in patients with higher NLR at 24 h post-poisoning than in patients with lower NLR. In the regression analysis, blood lactate was an independent predictor of severe poisoning but not of mortality, which agrees with a previous study [26].

Increased mortality in older patients with AOPP may be due to a decline in cholinergic function [27,28]. However, univariate and multivariate logistic regression analyses showed that age was not an independent predictor of poor prognosis in our cohort, and this result may be due to the small sample size.

Tallat et al. reported that serum caspase activity was a good predictor of outcome and mortality in patients with AOPP [4]; nonetheless, this marker is not used on a large scale because it is difficult to measure in the clinical setting. Farooqui et al. discussed the possibility of using latent class growth analysis to assess the relationship between vital signs and OP poisoning and proved that systolic blood pressure trajectories, heart and respiratory rate, and partial oxygen pressure were significantly associated with an increased risk of mortality in OP poisoning [8]. However, these authors did not identify a set of parameters that could be jointly used to guide clinical practice.

pChE activity is considered a good biomarker of exposure to OPs. However, there was no significant difference in the plasma concentrations of pChE between non-survivors and survivors (*p* = 0.078) in our cohort, and this result seems to corroborate a previous study [29]. pChE is not an accurate predictor of AOPP severity because it is not involved in cholinergic transmission in the nervous system [29] and, therefore, should be used in conjunction with other biomarkers.

The NLR reflects the interaction between host factors and the immune system in the disease state [30,31]. A retrospective study found that leukocyte and neutrophil counts and the NLR increased significantly in ventilated patients and non-survivors of acute pesticide poisoning, demonstrating that these parameters are useful for determining prognosis in patients with pesticide poisoning [32]. Our results suggest that the NLR can be used to predict severe poisoning and in-hospital mortality in patients with AOPP.

This study has strengths. First, poor prognosis included hypotension, ventilator support, cardiac arrest, and death. In contrast, previous studies are susceptible to bias because the only outcome evaluated was death. In our clinical practice, patients with severe AOPP associated with hemodynamic instability, coma, or respiratory failure are admitted to intensive care; therefore, the early assessment of the clinical status of patients is crucial to improve prognosis and reduce the total cost of hospitalization. Second, the NLR can be easily measured at the bedside by clinicians.

The study has limitations. First, the sample size was small. Second, retrospective studies are prone to bias by limiting comprehensive data collection. Third, the single-center nature of our study limits the applicability of the results to other healthcare settings or patient populations, and larger multicenter studies are warranted to confirm our findings.

## 5 Conclusion

High NLR is significantly associated with a poor prognosis of AOPP. Therefore, patients can be screened in time according to clinical symptoms and NLR values, so that medical staff can focus on the treatment and clinical progress of patients with poor prognosis. After stabilizing vital signs, AOPP patients with NLR >13 can be transferred to intensive care units and promptly receive ventilation support and appropriate treatment to improve prognosis and reduce total hospitalization cost.

## Appendix

**Table A1:** Assessment of collinearity in the multivariate model using the variance inflation factor

Variables	Variance inflation factor	
	Severe organophosphorus pesticide poisoning	Death
Age (years)	1.16	NA
Diabetes, *n* (%)	NA	1.60
Hypertension, *n* (%)	1.15	1.59
NLR	1.04	1.03
pChE	1.07	1.03
Amylase	1.07	NA
sCr	1.04	1.05
Lactic acid	1.09	NA

## References

[1] Mew EJ, Padmanathan P, Konradsen F, Eddleston M, Chang SS, Phillips MR, et al. The global burden of fatal self-poisoning with pesticides 2006–15: systematic review. J Affect Disord. 2017;219:93–104. 10.1016/j.jad.2017.05.002. 28535450

[2] Wu X, Xie W, Cheng Y, Guan Q. Severity and prognosis of acute organophosphorus pesticide poisoning are indicated by C-reactive protein and copeptin levels and APACHE II score. Exp Ther Med. 2016;11(3):806–10. 10.3892/etm.2016.2982. PMC4774328 26997996

[3] Khosravani H, Shahpori R, Stelfox HT, Kirkpatrick AW, Laupland KB. Occurrence and adverse effect on outcome of hyperlactatemia in the critically ill. Crit Care. 2009;13(3):R90. 10.1186/cc7918. PMC2717461 19523194

[4] Tallat S, Hussien R, Mohamed RH, Abd El Wahab MB, Mahmoud M. Caspases as prognostic markers and mortality predictors in acute organophosphorus poisoning. J Genet Eng Biotechnol. 2020;18(1):10. 10.1186/s43141-020-00024-y. PMC7152583 32281011

[5] Dungdung A, Kumar A, Kumar B, Preetam M, Tara RK, Saba MK. Correlation and prognostic significance of serum amylase, serum lipase, and plasma cholinesterase in acute organophosphorus poisoning. J Family Med Prim Care. 2020;9(4):1873–7. 10.4103/jfmpc.jfmpc_205_20. PMC7346941 32670933

[6] Dong N, Liu J, Wang Z, Gao N, Pang L, Xing J. Development of a practical prediction scoring system for severe acute organophosphate poisoning. J Appl Toxicol. 2020;40(7):889–96. 10.1002/jat.3950. 32030807

[7] Yuan S, Gao Y, Ji W, Song J, Mei X. The evaluation of acute physiology and chronic health evaluation II score, poisoning severity score, sequential organ failure assessment score combine with lactate to assess the prognosis of the patients with acute organophosphate pesticide poisoning. Medicine (Baltimore). 2018;97(21):e10862. 10.1097/MD.0000000000010862. PMC6392888 29794787

[8] Farooqui WA, Uddin M, Qadeer R, Shafique K. Trajectories of vital status parameters and risk of mortality among acute organophosphorus poisoning patients – a latent class growth analysis. BMC Public Health. 2020;20(1):1538. 10.1186/s12889-020-09637-x. PMC7552362 33046064

[9] Akilli NB, Yortanli M, Mutlu H, Gunaydin YK, Koylu R, Akca HS, et al. Prognostic importance of neutrophil-lymphocyte ratio in critically ill patients: short- and long-term outcomes. Am J Emerg Med. 2014;32(12):1476–80. 10.1016/j.ajem.2014.09.001. 25264245

[10] Afari ME, Bhat T. Neutrophil to lymphocyte ratio (NLR) and cardiovascular diseases: an update. Expert Rev Cardiovasc Ther. 2016;14(5):573–7. 10.1586/14779072.2016.1154788. 26878164

[11] Luo Y, Xia LX, Li ZL, Pi DF, Tan XP, Tu Q. Early neutrophil-to-lymphocyte ratio is a prognostic marker in acute minor stroke or transient ischemic attack. Acta Neurol Belg. 2020. 10.1007/s13760-020-01289-3. 32036555

[12] Ying HQ, Deng QW, He BS, Pan YQ, Wang F, Sun HL, et al. The prognostic value of preoperative NLR, d-NLR, PLR and LMR for predicting clinical outcome in surgical colorectal cancer patients. Med Oncol. 2014;31(12):305. 10.1007/s12032-014-0305-0. 25355641

[13] Omichi K, Cloyd JM, Yamashita S, Tzeng CD, Conrad C, Chun YS, et al. Neutrophil-to-lymphocyte ratio predicts prognosis after neoadjuvant chemotherapy and resection of intrahepatic cholangiocarcinoma. Surgery. 2017;162(4):752–65. 10.1016/j.surg.2017.05.015. 28688518

[14] Mandaliya H, Jones M, Oldmeadow C, Nordman II. Prognostic biomarkers in stage IV non-small cell lung cancer (NSCLC): neutrophil to lymphocyte ratio (NLR), lymphocyte to monocyte ratio (LMR), platelet to lymphocyte ratio (PLR) and advanced lung cancer inflammation index (ALI). Transl Lung Cancer Res. 2019;8(6):886–94. 10.21037/tlcr.2019.11.16. PMC6976360 32010567

[15] Eddleston M, Buckley NA, Eyer P, Dawson AH. Management of acute organophosphorus pesticide poisoning. Lancet. 2008;371(9612):597–607. 10.1016/S0140-6736(07)61202-1. PMC2493390 17706760

[16] Eddleston M. Patterns and problems of deliberate self-poisoning in the developing world. QJM. 2000;93(11):715–31. 10.1093/qjmed/93.11.715. 11077028

[17] Eddleston M. Novel clinical toxicology and pharmacology of organophosphorus insecticide self-poisoning. Annu Rev Pharmacol Toxicol. 2019;59:341–60. 10.1146/annurev-pharmtox-010818-021842. 30230960

[18] Vale A. Organophosphorus insecticide poisoning. BMJ Clin Evid. 2015;2015:2102. PMC4664131 26618560

[19] Nomura K, Narimatsu E, Inoue H, Kyan R, Sawamoto K, Uemura S, et al. Mechanism of central hypopnoea induced by organic phosphorus poisoning. Sci Rep. 2020;10(1):15834. 10.1038/s41598-020-73003-5. PMC7522229 32985607

[20] Naughton SX, Terry AV Jr. Neurotoxicity in acute and repeated organophosphate exposure. Toxicology. 2018;408:101–12. 10.1016/j.tox.2018.08.011. PMC6839762 30144465

[21] Chen KX, Zhou XH, Sun CA, Yan PX. Manifestations of and risk factors for acute myocardial injury after acute organophosphorus pesticide poisoning. Medicine (Baltimore). 2019;98(6):e14371. 10.1097/MD.0000000000014371. PMC6380665 30732172

[22] Karami-Mohajeri S, Abdollahi M. Mitochondrial dysfunction and organophosphorus compounds. Toxicol Appl Pharmacol. 2013;270(1):39–44. 10.1016/j.taap.2013.04.001. 23578477

[23] Binukumar BK, Bal A, Kandimalla R, Sunkaria A, Gill KD. Mitochondrial energy metabolism impairment and liver dysfunction following chronic exposure to dichlorvos. Toxicology. 2010;270(2–3):77–84. 10.1016/j.tox.2010.01.017. 20132858

[24] Hou YX, Liu SW, Wang LW, Wu SH. Physiopathology of multiple organ dysfunctions in severely monocrotophos-poisoned rabbits. Chem Biol Interact. 2017;278:9–14. 10.1016/j.cbi.2017.08.016. 28864276

[25] Sumathi ME, Kumar SH, Shashidhar KN, Takkalaki N. Prognostic significance of various biochemical parameters in acute organophosphorus poisoning. Toxicol Int. 2014;21(2):167–71. 10.4103/0971-6580.139800. PMC4170558 25253926

[26] Erfantalab P, Soltaninejad K, Shadnia S, Zamani N, Hassanian-Moghaddam H, Mahdavinejad A, et al. Trend of blood lactate level in acute aluminum phosphide poisoning. World J Emerg Med. 2017;8(2):116–20. 10.5847/wjem.j.1920-8642.2017.02.006. PMC5409231 28458755

[27] Gamage R, Wagnon I, Rossetti I, Childs R, Niedermayer G, Chesworth R, et al. Cholinergic modulation of glial function during aging and chronic neuroinflammation. Front Cell Neurosci. 2020;14:577–912. 10.3389/fncel.2020.577912. PMC7594524 33192323

[28] Gunduz E, Dursun R, Icer M, Zengin Y, Gullu MN, Durgun HM, et al. Factors affecting mortality in patients with organophosphate poisoning. J Pak Med Assoc. 2015;65(9):967–72. 26338743

[29] Jokanovic M. Neurotoxic effects of organophosphorus pesticides and possible association with neurodegenerative diseases in man: a review. Toxicology. 2018;410:125–31. 10.1016/j.tox.2018.09.009. 30266654

[30] Moore MM, Chua W, Charles KA, Clarke SJ. Inflammation and cancer: causes and consequences. Clin Pharmacol Ther. 2010;87(4):504–8. 10.1038/clpt.2009.254. 20147899

[31] Roxburgh CS, McMillan DC. Role of systemic inflammatory response in predicting survival in patients with primary operable cancer. Future Oncol. 2010;6(1):149–63. 10.2217/fon.09.136. 20021215

[32] Dundar ZD, Ergin M, Koylu R, Ozer R, Cander B, Gunaydin YK. Neutrophil-lymphocyte ratio in patients with pesticide poisoning. J Emerg Med. 2014;47(3):286–93. 10.1016/j.jemermed.2014.01.034. 24958695

